# Case report: Metaplastic carcinoma presenting as a breast abscess

**DOI:** 10.1186/1477-7800-3-23

**Published:** 2006-09-02

**Authors:** Corrine Wong, Chris Wright, Angela Colclough, Simon Marsh

**Affiliations:** 1 SHO in Surgery, Essex Rivers Healthcare NHS Trust, Department of Surgery, Colchester General Hospital, Turner Road, Colchester, Essex, CO4 5JL, UK; 2 SHO in Surgery, Department of Surgery, Essex Rivers Healthcare NHS Trust, Colchester General Hospital, Turner Road, Colchester, Essex, CO4 5JL, UK; 3 Consultant Pathologist, Department of Histopathology, Essex Rivers Healthcare NHS Trust, Colchester General Hospital, Turner Road, Colchester, Essex, CO4 5JL, UK; 4 Consultant Surgeon, Department of Surgery, Essex Rivers Healthcare NHS Trust, Colchester General Hospital, Turner Road, Colchester, Essex, CO4 5JL, UK

## Abstract

Metaplastic breast carcinoma (MBC) is a rare neoplasm containing a mixture of epithelial and mesenchymal elements. The epithelial component is usually ductal carcinoma but may include other variants of breast carcinomas including squamous carcinoma and osteogenic sarcoma. There is a relative paucity of data regarding such tumours. Metaplastic carcinoma carries a prognosis not dissimilar to that of comparable ductal carcinoma. This is the case of a 57 year old patient with MBC presenting with a breast abscess. A thorough literature search has not revealed any previous reports of MBC presenting as a breast abscess.

## Background

Metaplastic breast carcinoma (MBC) is a rare neoplasm containing a mixture of malignant epithelial and mesenchymal elements. The epithelial component is usually ductal carcinoma but may include other variants of breast carcinomas including squamous carcinoma. The mesenchymal component is usually composed of non-specific malignant spindle cells but may include differentiated sarcomas including fibrosarcoma, leiomyosarcoma and osteogenic sarcoma [1]. It represents as little as 0.02% of all breast malignancies [2]. There is a relative paucity of data regarding treatment options and prognosis. A thorough literature search (PUBMED and MEDLINE) has revealed a number of case reports where such tumours have presented as a breast lump, but this is the first report of MBC presenting as a breast abscess.

## Case history

A 57 year-old postmenopausal Caucasian woman with no significant past medical history presented with a four week history of rapid swelling, pain and erythema in her right breast. There was no history of trauma, and symptoms had not settled with oral antibiotics. She had never taken hormone replacement therapy. She had a low grade pyrexia, but was otherwise systemically well.

On examination, her entire right breast was erythematous, warm and swollen, with fluctuance and pointing in the outer lower quadrant. Otherwise, the breast was firm and non-fluctuant. The right nipple had been inverted since her symptoms began. Initial investigations revealed raised inflammatory markers and a neutrophillia. She also had a raised alkaline phosphatase of 615.

An initial diagnosis of a breast abscess was made, possibly secondary to an inflammatory carcinoma, and needle aspiration was attempted, although this did not yield pus. She was commenced on intravenous antibiotics and an ultrasound was arranged, which showed multiple loculations containing thick pus.

As the patient was in considerable distress, she underwent incision and drainage of this collection. The abscess was drained, although the cavity wall was noted to be suspicious and open biopsies were taken. Histology showed "metaplastic carcinoma with features of osteogenic sarcoma." She then underwent staging CT and bone scans. CT demonstrated multiple small calcified nodules in both lungs consistent with metastatic deposits. Bone scan did not show any skeletal lesions.

The patient underwent three cycles of neoadjuvant chemotherapy with ifosphamide and adriamycin, but this was poorly tolerated, and had only a modest effect on the primary tumour and metastatic lesions. Upon cessation of chemotherapy, there was rapid re-growth in the primary lesion, and the patient was admitted for surgery, in an attempt to achieve local disease control. She underwent radical mastectomy, axillary clearance and primary reconstruction with a latissimus dorsi flap. Recovery was uneventful.

Histology described a large tumour (16 × 11 × 8 cm), invading the breast and muscle, with extensive vascular and lymphatic invasion and confirmed the diagnosis of metaplastic breast carcinoma with osteogenic sarcoma predominating. All sixteen lymph nodes removed were involved. Surgical margins were at least 1 cm in all directions, and Paget's disease of the nipple was noted. The tumour was negative for oestrogen and progesterone receptors.

Two months later, she developed a new mass in the infra-axillary region on the right, which rapidly enlarged. She was given palliative chemotherapy with etopiside and carboplatin, during which she was admitted with neutropenic sepsis, from which she recovered. She was, however, re-admitted three weeks later with deterioration in her condition, and died in hospital a week later. The time from first presentation to her death was almost exactly eight months.

## Discussion

The presentation of malignancy as a breast abscess is well described [3], however, to our knowledge, this is the first reported case of MBC presenting in this way.

MBC is a very rare neoplasm. 3 recently published series over 13, 11 and 16 years yielded only 21, 19 and 43 patients respectively [4-6]. Even these have conflicting conclusions regarding the most important prognostic indicators and treatment options. Gibson et al [4] say that MBC tends to present in an advanced stage and often recurs locally, but survival is similar to that of adenocarcinomas of comparable stage, and hence treatment should follow similar principles. Al Sayed et al [5], however, state that MBC is aggressive with a poor outcome. They also found that an important factor in determining outcome is tumour size.

The mesenchymal element involved would seem to be important in determining outcome [2]. In the case described, this was osteogenic sarcoma (OS). Pure extra-osseous OS is an extremely rare neoplasm. In fact, one of the largest series described only sixty cases over forty years [7]. Of these, only 27% were sited in the trunk or thorax. Most patients with OS of the breast invariably present with a breast lump.

Extra-osseous OS differs from primary bone osteosarcoma importantly in its response to chemotherapy. Complete pathologic response rates of 25–60% are achievable for bone osteosarcoma, however with extra-osseous OS response rates are only 19% at best [8].

The prognosis for pure extra-osseous OS is similar to other forms of high-grade soft tissue sarcoma. Those with metastases at the time of diagnosis have a median survival of only eight months, with a disease-specific 5-year survival of only 10% [7].

## Conclusion

Metaplastic carcinoma of the breast is an extremely rare malignancy, which, to our knowledge, has never before been reported as presenting as a breast abscess. As pure extra-osseous osteogenic sarcoma is much more chemoresistant compared with primary bone osteosarcoma, one can surmise that metaplastic carcinomas with a predominating OS element will behave similarly indicating that treatment options are limited.

**Figure 1:**
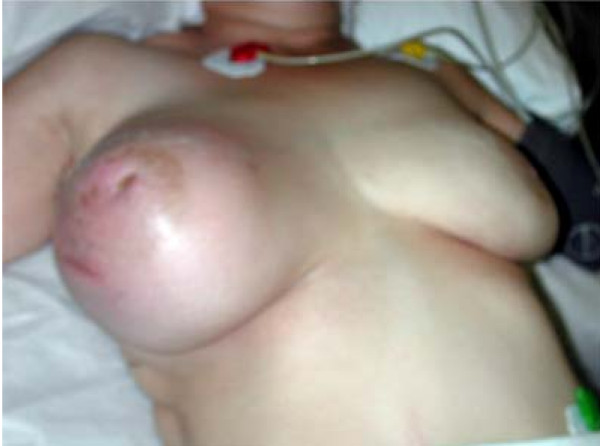
Right breast following chemotherapy at time of surgery.

**Figure 2:**
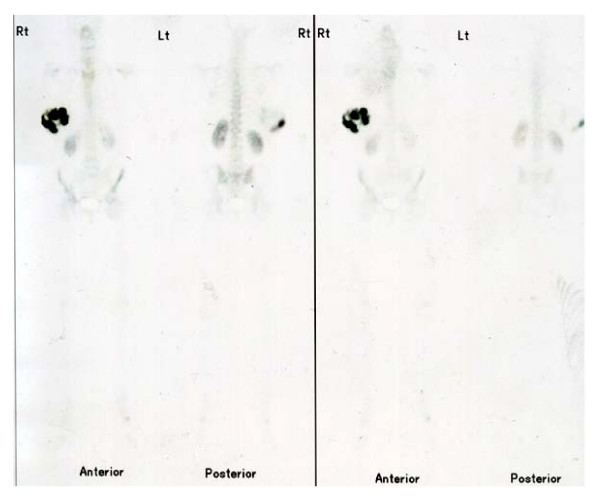
Bone scan showing isotope uptake in right breast.

**Figure 3:**
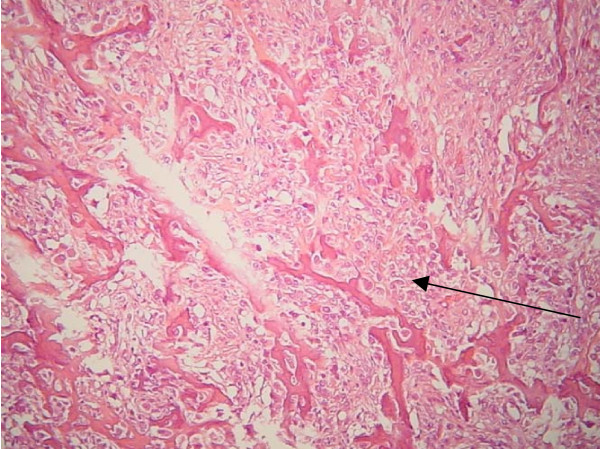
Histopathology of tumour demonstrating osteogenic sarcoma.

## References

[B1] Beatty JD, Atwood M, Tickman R, Reiner M (2006). Metaplastic breast cancer: clinical significance. Am J Surg.

[B2] Alam K, Maheshwari V, Harris H, Mehdi G (2003). An unusual case of metaplastic breast carcinoma (sarcomatoid variant). Indian J Surg.

[B3] Cappellani A, Di Vita M, Zanghi A (2004). A pure squamous cell breast carcinoma presenting as a breast abscess: case report and review of literature. Ann Ital Chir.

[B4] Gibson GR, Qian D, Ku JK, Lai LL (2005). Metaplastic breast cancer: clinical features and outcomes. Am Surg.

[B5] Al Sayed AD, El Weshi AN, Tulbah AM, Rahal MM, Ezzat AA (2006). Metaplastic carcinoma of the breast clinicalpresentation, treatment results and prognostic factors. ActaOncol.

[B6] Dave G, Cosmatos H, Do T, Lodin K, Varshney D (2005). Metaplastic carcinoma of the breast: a retrospective review. Int J Radiat Oncol Biol Phys Epub.

[B7] Ahmad S, Patel S, Ballo M (2002). Extraosseous Osteosarcoma: Response to treatment and long term outcome. Journal of Clinical Oncology.

[B8] Bacci G, Picci P, Ferrari S (1993). Primary chemotherapy and delayed surgery for nonmetastatic osteosarcoma of the extremities: Results in 164 patients preoperatively treated with high doses of methotrexatefollowed by cisplatin and doxorubicin. Cancer.

